# Gut Microbiota Modulation in Type 2 Diabetes and Cardiometabolic Risk: A Systematic Review

**DOI:** 10.7759/cureus.92020

**Published:** 2025-09-10

**Authors:** Umer Qureshi, Ali Bajwa, Zohaib Aslam, Abena Aggrey, Usama Hassan Nawaz, Qurrat Ul Ain

**Affiliations:** 1 General and Colorectal Surgery, The Royal Oldham Hospital, Oldham, GBR; 2 Acute Medicine, Royal Derby Hospital, Derby, GBR; 3 Medicine, Life Care Children Hospital, Lahore, PAK; 4 General Medicine, Mid Cheshire NHS, Cheshire, GBR; 5 Neurorehabilitation, Queen Mary’s Hospital, London, GBR; 6 Medicine, Rawal General and Dental Hospital, Islamabad, PAK

**Keywords:** cardiometabolic risk, glycemic control, gut microbiota, prebiotics, probiotics, type 2 diabetes mellitus (t2dm)

## Abstract

Cardiometabolic complications related to type 2 diabetes mellitus (T2DM) are often due to changes in the gut microbiota. The review analyzed studies looking at the effects of probiotics, prebiotics, high-fiber diets, and fecal microbiota transplantation (FMT) on glucose levels and heart and metabolic health in individuals either having T2DM or being at risk. The review followed the Preferred Reporting Items for Systematic Reviews (PRISMA) guidelines. The literature was searched using text terms and controlled vocabulary, employing Boolean operators "AND," "OR," and various combinations across PubMed, Embase, and the Cochrane Library. Open-access, full-text English papers from 2005 to 2025, including those authored by people, were searched. The quality was assessed using the Risk of Bias 2.0 (RoB 2.0) tool, and the evidence was appraised using the Grading of Recommendations, Assessment, Development, and Evaluation (GRADE) approach. Fifteen randomized controlled trials (RCTs) were analyzed for methodological quality, with three categorized as having a high risk of bias (RoB). The GRADE tool categorized two high RoB RCTs as "low quality." However, two RCTs had low RoB and were classified as "high quality." Ten RCTs had uncertain RoB, lowering the evidence by one point to "moderate quality." A comprehensive review of RCTs was conducted to assess outcomes related to glycemic parameters (e.g., glycated hemoglobin (HbA1c), fasting glucose), lipid profiles, inflammatory markers, anthropometric measures, and gut microbiota composition. Interventions included probiotic and prebiotic supplementation, high-fiber or Mediterranean-style diets, and FMT. Probiotic yogurt containing Lactobacillus acidophilus and Bifidobacterium lactis significantly improved lipid profiles by reducing low-density lipoprotein cholesterol (LDL-C) and total cholesterol. High-fiber diets consistently lowered fasting blood glucose, HbA1c, triglycerides, and LDL-C while elevating high-density lipoprotein cholesterol (HDL-C) and beneficial short-chain fatty acid (SCFA)-producing bacteria. Anti-inflammatory effects were observed across interventions, notably with probiotics and polyphenol-rich Mediterranean diets, which reduced tumor necrosis factor-alpha (TNF-α), interleukin-6 (IL-6), and other inflammatory cytokines. The Green-Mediterranean diet significantly improved weight, insulin resistance, and Framingham risk scores. Novel mechanisms involving SCFAs and bile acid metabolism were also identified as key modulators of host metabolic response. Microbiota-based interventions offer promising avenues for glycemic control and cardiometabolic risk reduction in patients with T2DM.

## Introduction and background

The gut microbiota of various microbes in the digestive organs is needed for good metabolism, the immune system, and health. The prevalence of diabetes, obesity, dyslipidemia, and hypertension (HTN), which are similar in nature, is greatly influenced by it [1]. An imbalance in gut microbes (dysbiosis) can make blood glucose levels change, cause major inflammation, and eventually lead to insulin-resistant states in the body [2].

Improper levels of gut bacteria can trigger type 2 diabetes mellitus (T2DM), which can affect other body systems. Having an unbalanced gut (called dysbiosis) causes endotoxemia and continual inflammation, makes the lining of the gut less intact, and leads to lipopolysaccharide (LPS) released by harmful bacteria, resulting in more inflammation, less response to insulin in the body, and less sensitivity to insulin [3]. Not enough short-chain fatty acids (SCFAs) exist if bacteria in the gut break down less dietary fiber, resulting in glucose intolerance. When bile metabolism is disrupted, the farnesoid X receptor (FXR) and Takeda G protein-coupled receptor 5 (TGR5) become less sensitive, which influences poor glucose regulation, rises in gluconeogenesis, and less insulin secretion [4]. Some bacteria found in the intestine make the chemical trimethylamine N-oxide (TMAO), which is associated with insulin resistance and a higher chance of heart disease in T2DM [5]. Intestinal function and immune system changes can lead to more metabolic troubles. Because the mucosal layer weakens, endotoxins can enter the body and trigger the immune response. Because of this reaction, tumor necrosis factor-alpha (TNF-α) and interleukin-6 (IL-6) are released, which interfere with insulin’s effects and the work of β-cells [6]. Handling T2DM may comprise balancing gut bacterial populations, introducing probiotics and prebiotics through food, and performing fecal microbiota transplantation (FMT), all to improve gut well-being, stimulate SCFA production among gut bacteria, and bring down inflammation [7].

Cardiometabolic risk rises because of the many abnormalities linked to gut dysbiosis. Suppose bacteria cause inflammation or endotoxemia in the body. In that case, it becomes easy for toxins such as LPS to get into the blood, causing inflammation and affecting both insulin resistance and the endothelial cells [8]. A shortage of SCFAs in the body due to these bacteria may disrupt glucose and lipid metabolism and lead to metabolic syndrome. SCFAs play a role in avoiding insulin resistance and liver fat, and losing them can cause more trouble with these conditions [9].

When atherogenic pathways in the gut break down choline and carnitine, they become TMAO, which helps to form foam cells and destabilizes plaque, increasing the possibility of atherosclerosis [10]. Issues with secondary bile acid production can change cholesterol levels and cause more inflammation in blood vessels, which increases heart-related risks. Dysbiosis also leads to improper levels of adipose tissue, leading to obesity-linked metabolic disorders [11]. Toxins produced by microbes in uremia cause damage to blood vessels and cell walls, leading to HTN. Some microbial groups that help plants and animals include nitrogen-fixing bacteria, mycorrhizae, and various gut microbes. Imbalanced gut microbes can worsen metabolic problems, leading to further changes in gut microbes [12]. Targeted steps like probiotics, prebiotics, following specific diets, or microbiota-targeted therapies could help reduce inflammation, restore balance to the microbiome, and improve cardiometabolic results [13].

The gut microbiota significantly influences metabolic health through pathways involving insulin sensitivity, lipid regulation, and systemic inflammation. While alterations in gut bacteria have been linked to disease development, the clinical application of microbiota-based therapies remains inconsistent. In this review, we focus on randomized controlled trials (RCTs) to provide the highest level of clinical evidence on gut microbiota interventions in T2DM and cardiometabolic risk. Importantly, beyond summarizing established mechanisms such as SCFAs, bile acids, and TMAO, this review highlights critical knowledge gaps, particularly in translating laboratory findings into clinical practice and identifying which patient populations may benefit most, thus guiding future research in metabolic disease control.

## Review

This review followed the requirements established by the Preferred Reporting Items for Systematic Reviews and Meta-Analyses (PRISMA) guidelines [14]. The research question was formulated using the Population, Intervention, Comparator, and Outcome (PICO) framework, as shown in Table 1 [15].

**Table 1:** PICO framework T2DM, type 2 diabetes mellitus; PICO, Population, Intervention, Comparator, and Outcome

Concept	Text words	Controlled vocabulary
Population/Problem: Adults with T2DM	“Type 2 Diabetes” OR “Type II Diabetes Mellitus” OR “T2DM” OR “Insulin Resistance”	"Diabetes Mellitus, Type 2" [MeSH] OR "Insulin Resistance" [Mesh]
Intervention: Gut microbiota modulation (e.g., probiotics, prebiotics, dietary fibre)	“Gut microbiota modulation” OR “Probiotics” OR “Prebiotics” OR “Synbiotics” OR “fecal transplant”	"Gastrointestinal Microbiome" [Mesh] OR "Probiotics" [Mesh] OR "Prebiotics" [Mesh] OR "Fecal Microbiota Transplantation" [Mesh]
Comparison: Standard care or placebo	“No modulation” OR “Placebo” OR “Standard treatment”	"Placebo" [Mesh] "Standard treatment" [Mesh]
Outcome: Improved glycemic control and reduced cardiometabolic risk	“Cardiometabolic risk” OR “Lipid profile” OR “Blood pressure” OR “Cardiovascular risk” OR “Glucose metabolism”	"Cardiovascular Diseases" [Mesh] OR "Metabolic Syndrome" [Mesh] OR "Insulin Resistance" [Mesh] OR "Blood Glucose" [Mesh]

How does gut microbiota modulation through probiotics, prebiotics, or dietary fiber affect glycemic control and cardiometabolic risk in patients with T2DM?

Search strategy and search terms

Searches were conducted using combinations of keywords and controlled vocabulary related to “type 2 diabetes mellitus (T2DM),” “probiotics and dietary fiber,” “gut microbiota,” and glycemic control, such as “lipid profile,” “metabolic outcomes,” and “inflammatory outcomes.” Boolean operators “AND” and “OR” were used to connect relevant terms across databases, including PubMed, Embase, and the Cochrane Library. The search focused on text words and controlled vocabulary (e.g., Medical Subject Headings (MeSH) terms) to ensure comprehensive coverage. Limiters were applied to retrieve only open-access, full-text articles published in English between 2014 and 2024 and studies involving human participants.

Search String

("type 2 diabetes mellitus" OR "T2DM" OR "diabetes mellitus, type 2") AND ("probiotics" OR "probiotic yogurt") AND ("prebiotics" OR "high-fiber diet") AND ("gut microbiota" OR "fecal microbiota transplantation [FMT]" OR "bacteriophage") AND ("glycemic control" OR "blood glucose" OR "glycated hemoglobin [HbA1c]" OR "insulin sensitivity" OR "glucose metabolism") AND ("lipid profile" OR "cholesterol" OR "triglycerides" OR "low-density lipoprotein [LDL]" OR "high-density lipoprotein [HDL]") AND ("inflammation" OR "tumor necrosis factor-alpha [TNF-α]" OR "interleukin-6 [IL-6]" OR "C-reactive protein [CRP]") AND ("short-chain fatty acids [SCFAs]" OR "acetate" OR "butyrate").

Study selection criteria for systematic review

Table 2 explains the criteria for study inclusion in the systematic review.

**Table 2:** Inclusion and exclusion criteria for the review

Criteria type	Criteria
Inclusion	Only experimental studies with varying sample sizes.
	Adult patients (≥18 years) of either gender with type II diabetes or cardiometabolic risk.
	Intervention: Gut microbiota modulation treatment.
	Outcomes: Focused on glycemic control and lipid profile.
	Study characteristics: Open-access, published in the last two decades (2005-2025), in English, and available in full text.
Exclusion	Non-experimental study designs (cohort, case-control, observational studies, case reports, case series, conference abstracts, editorials, letters, review papers, and meta-analyses).
	Studies involving teenagers, children, or animals.
	Studies published before 2005.
	Studies in which patient outcomes were unrelated to gut microbiota modulation in type II diabetes or cardiometabolic risk.
	Studies with restricted data access or incomplete analysis.

Study selection process

The initial screening involved two independent reviewers reading the articles’ titles and abstracts. Then, the two independent reviewers conducted a full-text review by comprehensively reading the articles. In case of disagreement, a consensus was developed [16]. The review included only those studies available in full text that met the inclusion criteria.

Methodological quality assessment

The risk of bias (RoB) was assessed using the Cochrane Risk of Bias 2.0 (RoB 2.0) tool, which divided studies into three categories: high, low, and some concerns regarding RoB. The Grading of Recommendations, Assessment, Development, and Evaluation (GRADE) approach was employed to grade trial results into high quality, moderate quality, low quality, and very low quality [17].

Data extraction and synthesis

A datasheet was created to collect details from the included studies to synthesize findings. This review encompassed basic information such as study design, demographic characteristics, and outcome-related details, including author/year, objectives, study design/population characteristics, intervention protocol, outcomes measured, and complications. A thematic analysis using an inductive, data-driven approach was employed to analyze the datasheet [18]. An iterative approach was then applied for a further in-depth review and convergence of results [19]. Finally, studies were critically analyzed to synthesize the evidence, ensuring evidence-based practice.

Ethical consideration

**Figure 1:**
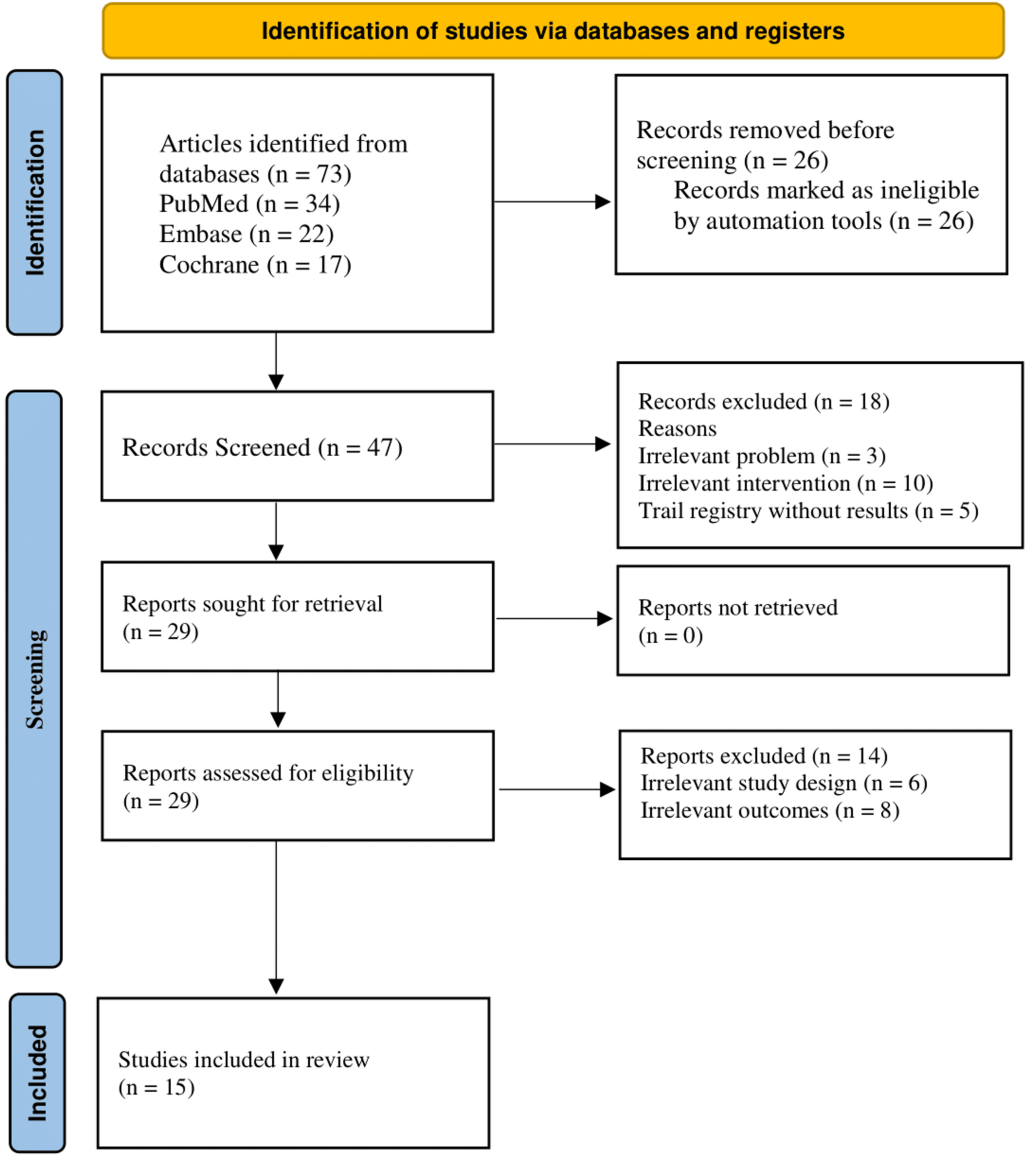
PRISMA chart PRISMA, Preferred Reporting Items for Systematic Reviews and Meta-Analyses

The review adhered to the Declaration of Helsinki to meet ethical standards throughout the study. There are no conflicts of interest among the reviewers. The search strategy was performed with specific keywords to ensure reproducibility. The study will be published in a peer-reviewed medical journal to disseminate findings publicly while maintaining confidentiality and anonymity. The study met the PRISMA guidelines (Figure 1).

Results

This systematic review was conducted in accordance with PRISMA guidelines. A comprehensive search of PubMed, Embase, and the Cochrane Library was performed for studies published between 2005 and 2024. Search terms combined keywords and controlled vocabulary for “type 2 diabetes mellitus (T2DM),” “probiotics,” “prebiotics/dietary fiber,” “gut microbiota,” and metabolic outcomes (glycemic control, lipid profile, inflammatory markers, and SCFAs), connected with Boolean operators “AND” and “OR.” The initial search identified 73 records, of which 26 duplicates were removed; 47 records were screened, 18 excluded by title/abstract, and 14 excluded after full-text review, leaving 15 eligible studies for quality assessment.

Cochrane Risk of Bias Assessment of Studies Included in Systematic Review

Table 3 presents the assessment of risk of bias for the included studies. The assessment checks for biases in selection, performance, detection, and reporting. It reviews the design and analysis methods of the studies and shows where their RoB is high, low, or not assessable. The outcomes let the reviewers know how strongly to rely on the findings and the proof gathered.

**Table 3:** Cochrane ROB RoB, risk of bias

Author/year	Randomization process	Deviations from intended intervention	Missing outcome data	Outcome measurement	Selection of reported results	Overall quality (RoB)
Tonucci et al., 2016 [20]	Low	Some concerns	Low	Low	Low	Moderate
Ejtahed et al., 2011 [21]	Low	Low	Some concerns	Low	Low	Moderate
Lestari et al., 2019 [22]	Low	Some concerns	Low	Some concerns	Low	Moderate
Zhao et al., 2018 [23]	Low	Low	Low	Low	Some concerns	Moderate
Chen et al., 2023 [24]	Low	Low	Some concerns	Low	Low	Moderate
Wan et al., 2019 [25]	Low	Some concerns	Low	Some concerns	Low	Moderate
Manrique et al., 2021 [26]	Some concerns	Some concerns	Low	Some concerns	Some concerns	Poor
Gómez-Pérez et al., 2024 [27]	Low	Low	Some concerns	Low	Low	Moderate
Ramos-Romero et al., 2020 [28]	Low	Low	Some concerns	Low	Low	Moderate
Vijay et al., 2021 [29]	Low	Low	Low	Low	Low	High
Rinott et al., 2022 [30]	Low	Some concerns	Some concerns	Low	Some concerns	Poor
Gao et al., 2024 [31]	Low	Low	Low	Low	Low	High
Ahrens et al., 2021 [32]	Low	Some concerns	Low	Low	Some concerns	Moderate
Ranaiyo et al., 2022 [33]	Low	Low	Some concerns	Low	Low	Moderate
Djekic et al., 2020 [34]	Low	Some concerns	Some concerns	Low	Some concerns	Poor

Tonucci et al. (2016), Ejtahed et al. (2011), Lestari et al. (2019), Zhao et al. (2018), Chen et al. (2023), Wan et al. (2019), Gómez-Pérez et al. (2024), Ramos-Romero et al. (2020), Ahrens et al. (2021), and Ranaiyo et al. [20-25,27,28,32,33] were included in the review. While Vijay et al. (2021) and Gao et al. (2024) were rated as high quality overall, they had low risk across all domains, indicating strong methodological rigor [29,31]. In contrast, Manrique et al. (2021) and Djekic et al. (2020) presented multiple domains with some concerns, particularly in deviations, outcome measurement, and reporting, leading to an overall poor-quality rating [26,34]. Similarly, Rinott et al. (2022) also had poor quality, mainly due to issues in deviation, missing data, and reporting bias [30]. Fifteen RCTs were analyzed for methodological quality, with three categorized as having high ROB. The GRADE tool categorized two high ROB RCTs as "low quality." However, two RCTs had low ROB and were classified as "high quality." Ten RCTs had uncertain ROB, lowering the evidence by one point to "moderate quality."

Characteristics and Findings of Studies Included in the Review

Table 4 describes the characteristics and findings of studies included in the review. It includes author/year, objectives, study design and population characteristics, intervention protocol, measured outcomes, and clinical implications.

**Table 4:** Summary of findings included in review T2D: type 2 diabetes mellitus, RCT: randomized controlled trial, FMT: fecal microbiota transplantation, CFU: colony-forming units, HbA1c: hemoglobin A1c, FBG: fasting blood glucose, TC: total cholesterol, LDL-C: low-density lipoprotein cholesterol, HDL-C: high-density lipoprotein cholesterol, TG: triglycerides, SCFAs: short-chain fatty acids, TNF-α: tumor necrosis factor-alpha, IL-10: interleukin-10, GLP-1: glucagon-like peptide 1, PYY: peptide YY, HOMA-IR: homeostasis model assessment of insulin resistance, OGTT: oral glucose tolerance test, MCP-1: monocyte chemoattractant protein-1, HAMA: Hamilton anxiety rating scale, HAMD: Hamilton depression rating scale, hs-CRP: high-sensitivity C-reactive protein, TXB₂: thromboxane B2, HV: high variance, Rd: rate of glucose disappearance, BMI: body mass index, MetS: metabolic syndrome, PERMANOVA: permutational multivariate analysis of variance, MED: Mediterranean, CMR: cardiometabolic risk, IHD: ischemic heart disease, TMAO: trimethylamine N-oxide.

Author/Year	Objectives	Study design/population characteristics	Intervention protocol	Measured outcome	Clinical implications
Tonucci et al., 2016 [20]	The study explores the impact of probiotics (Lactobacillus acidophilus La-5 and Bifidobacterium animalis subsp. lactis BB-12) on glycemic control, lipid profile, inflammation, oxidative stress, and SCFAs in T2DM patients.	Randomized, double-blind, placebo-controlled trial. 50 T2DM patients (aged 35-60 years, BMI <35 kg/m²) randomized into probiotic (n=25) and control (n=25) groups. 45 completed the study (90% adherence).	Probiotic group: 120 g/day fermented goat milk containing L. acidophilus La-5 and B. animalis BB-12 (10⁹ CFU/d each) for 6 weeks. Control group: Conventional fermented milk (no probiotics). Both groups maintained their usual diet and lifestyle.	Glycemic control: Significant reduction in fructosamine (-9.91 mmol/L; p=0.04) and trend for HbA1c (-0.67%; p=0.06) in probiotic group. Lipid profile: Probiotic group prevented increases in TC and LDL-C (p=0.04 and p=0.03 vs. control). Inflammation: TNF-α and resistin decreased in both groups (p<0.05); IL-10 decreased only in the control group (p<0.001). SCFAs: Acetic acid increased in both groups (p<0.01). No change in oxidative stress markers.	Probiotics (L. acidophilus La-5 and B. lactis BB-12) could be a supportive therapy for T2DM management, particularly for glycemic and lipid control.
Ejtahed et al., 2011 [21]	Investigate the effects of probiotic yogurt on lipid profile in T2DM individuals.	Randomized, double-blind controlled trial. 60 participants (23 males, 37 females) with T2DM and LDL-C >2.6 mmol/L. Age 30-60 years, BMI <35 kg/m².	Probiotic group: 300 g/day yogurt containing L. acidophilus La-5 and B. lactis BB-12. Control: 300 g/day conventional yogurt. Duration: 6 weeks.	Lipid profile: TC decreased by 4.54% in the probiotic group (P=0.008). LDL-C decreased by 7.45% (P=0.004). HDL-C and TG: no significant changes. Atherogenic indices: TC:HDL-C and LDL-C:HDL-C ratios significantly decreased (P=0.027 and P=0.033).	Probiotic yogurt may improve cardiovascular risk factors in T2DM by reducing TC and LDL-C. Further long-term studies are needed.
Lestari et al., 2019 [22]	To investigate the effects of probiotic yogurt (L. acidophilus La-5 and B. lactis BB-12) and conventional yogurt on FBG and lipid profiles in T2DM patients.	Randomized, double-blind controlled clinical trial. 38 T2DM patients (30-60 years) from Yogyakarta, Indonesia.	Intervention: 100 mL/day probiotic yogurt (L. acidophilus La-5 10⁸ CFU/g + B. lactis BB-12 10⁶ CFU/g). Control: 100 mL/day conventional yogurt (S. thermophilus + L. bulgaricus).	FBG: significant reduction in control (153→114 mg/dL, p=0.028). Non-significant in probiotic (139→126 mg/dL, p=0.173). Lipid profile: HDL-C increased in both groups (control: 37.75→41.80 mg/dL, p=0.011; probiotic: 36.60→41.30 mg/dL, p=0.001). TC increased in both (p<0.001). LDL/TG: no significant changes. Weight and BMI decreased (p<0.05).	HDL-C improvement: Both yogurts may benefit cardiovascular health. Glycemic control: conventional yogurt showed superior FBG reduction.
Zhao et al., 2018 [23]	Investigate how dietary fibers selectively promote gut bacteria that produce SCFAs and alleviate T2DM.	Open-label, parallel-group randomized trial (GUT2D study). 43 adults with T2DM (27 high-fiber, 16 control).	High-fiber: whole grains, traditional Chinese medicinal foods, prebiotics (WTP diet) for 84 days. Control: standard care + acarbose.	HbA1c: greater reduction in high-fiber group (89% <7% vs. 50% control; P<0.01). SCFAs: increased butyrate (P<0.05) and acetate. Gut hormones: higher GLP-1 (P<0.05) and PYY. Microbiota: 15 SCFA-producing strains promoted; negative responders declined. Fecal pH was reduced (P<0.05).	Targeted dietary fiber interventions can modulate gut microbiota to manage T2DM. SCFA-producing bacteria may serve as biomarkers. Personalized high-fiber diets could restore microbial ecosystem services.
Chen et al., 2023 [24]	To investigate the effects of a high-fiber diet on gut microbiota, serum metabolism, and mood in T2DM patients.	Randomized, open-label, parallel-group trial. 17 T2DM patients (HbA1c 6.5%-12.0%).	Control: standard T2DM diet. Treatment: high-fiber diet + acarbose (100 mg 3x/day).	Glucose homeostasis: ↓FBG (p<0.05) and HbA1c (p<0.001). ↑ Insulin and C-peptides (p<0.05). Lipid metabolism: ↓TC, TG, LDL-C (p<0.01); ↑HDL-C (p<0.05). Inflammation: ↓IL-1β, IL-6, TNF-α, MCP-1. Mood: ↓HAMA/HAMD scores. Microbiota: ↑ Lactobacillus, Bifidobacterium, Akkermansia; ↓ Desulfovibrio, Klebsiella; ↓ Firmicutes/Bacteroidota ratio.	High-fiber diets may enhance glycemic and lipid control. Dietary fiber could mitigate T2DM-related psychiatric comorbidities via the gut-brain axis. Fiber interventions may restore microbial balance.
Wan et al., 2019 [25]	To investigate the effects of dietary fat on gut microbiota, fecal metabolites, and cardiometabolic risk factors in healthy adults.	Randomized controlled-feeding trial. 217 healthy adults (18-35 years; BMI <28; 52% women).	Diets: Lower fat (20% fat, 66% carb), moderate fat (30% fat, 56% carb), higher fat (40% fat, 46% carb). Isocaloric with fixed protein (14%) and fiber (~14 g/day).	Metabolites: ↓ SCFAs in higher fat (p<0.001), ↑ pro-inflammatory metabolites. ↑ hs-CRP and TXB₂. Gut microbiota: ↑ α-diversity and beneficial taxa in lower fat; adverse shifts in higher fat.	Higher-fat diets may promote gut dysbiosis, reduce SCFAs, and increase inflammation. Lower-fat diets support metabolic health.
Manrique et al., 2021 [26]	Investigate dynamics of gut bacteriophages in MetS patients before/after FMT and associations with clinical outcomes.	MetS subjects: male, Caucasian, obese (BMI ≥30), n=9 (3 controls, 6 treatment). Healthy donors: lean, metabolically healthy, n=5.	Control: autologous FMT (self-stool, n=3). Treatment: allogenic FMT (n=6, responders/non-responders based on glucose disappearance rate).	FMT reduced recipient-unique phages (35% vs. 80% controls, p<0.0001). Specific phage groups enriched in responders correlated with improved Rd (p<0.05). Responders clustered closer to donors (PERMANOVA p=0.003).	Phage restructuring post-FMT may contribute to metabolic improvements. Specific phages may serve as biomarkers. Highlights the role of phages in gut microbiota modulation.
Gómez-Pérez et al., 2024 [27]	Evaluate effects of FMT from lean donors and probiotic supplementation on insulin sensitivity and glycemic control in T2DM with high insulin resistance.	Phase II, randomized, single-blind, parallel-arm trial. 21 adults (30-70 years) with T2D on metformin; BMI 30-40; HOMA-IR >2.	1) FMT: single oral lyophilized fecal microbiota from lean donors (BMI <25). 2) Probiotic: L. delbrueckii spp. bulgaricus LB-14 (25×10⁹ CFU/day). 3) Placebo: inert capsules.	Glycemic control: no significant changes in HbA1c, HOMA-IR, or OGTT. Mild HbA1c increase in FMT (+0.25%; p=0.041). The probiotic group reduced uric acid (-0.5 mg/dL; p=0.037). Microbiota: donor species increased in FMT; no overall diversity changes.	FMT and probiotics did not improve insulin sensitivity or glycemic control. Probiotics may modestly reduce uric acid, benefiting metabolic health.
Ramos-Romero et al., 2020 [28]	Investigate effects of grape pomace supplementation on gut microbiota and cardiometabolic markers.	Randomized cross-over trial. 49 subjects (55% male, mean age 42.6) with ≥2 metabolic syndrome factors.	8 g/day GP for 6 weeks, followed by a control period (usual diet). No placebo; subjects maintained their usual diet.	Insulin: significant reduction in responders (-40%, p<0.00001), increase in non-responders (+82%). Microbiota: no major phyla changes; decreased Lactobacillales trend (p=0.059). ↑ Bacteroides in GP-NR (p<0.01). SCFAs: reduced isovaleric acid (p<0.05).	GP may improve insulin sensitivity in some individuals; effects are not mediated by major gut microbiota changes. Species-level studies needed.
Vijay et al., 2021 [29]	Investigate the role of diet (omega-3 and fiber) in gut microbiome-CVD association and effects on CVD risk markers.	Two-arm randomized dietary intervention (open trial, no placebo). 70 healthy participants (mean age 65.2±9.3, 85.7% women, BMI 26.8±4.3).	Fiber arm: 20 g/day inulin (10 g twice daily). Omega-3: 500 mg/day (165 mg EPA + 110 mg DHA). Duration: 6 weeks. Compliance monitored.	Novel CVD markers: decreases in GlycA (fiber: β=-0.58, P=0.02; omega-3: β=-0.66, P=0.007) and ceramide ratios. Traditional markers: BP, cholesterol, LDL-C, VLDL-C, IL-4, and TNF-α decreased (P<0.05). Microbiome/SCFAs: Bifidobacterium and Coprococcus 3 increased; SCFAs mediated 54-77% of microbiome effect on CVD markers.	Fiber/omega-3 interventions can modulate gut microbiome and SCFA production, improving cardiovascular health. Supports targeting microbiota for CVD prevention.
Rinott et al., 2022 [30]	Investigate effects of MED and green-MED diets on gut microbiome and cardiometabolic health.	RCT (DIRECT-PLUS). 294 participants with abdominal obesity/dyslipidemia (88% male, mean age 51, BMI 31).	Groups: HDG, MED, Green-MED. HDG: standard nutrition + activity. MED: calorie-restricted MED diet + activity. Green-MED: MED + 3-4 cups green tea/day + 100 g Mankai + reduced red/processed meat.	Microbiome: Green-MED-induced compositional shifts (PERMANOVA P=0.01), enriched Prevotella, reduced Bifidobacterium. Cardiometabolic: greatest weight loss (-6.5% vs. -5.4% MED, -1.58% HDG), improved Framingham risk, waist, and HOMA-IR. Microbiome mediated 12% weight loss, 18% risk reduction.	The green-MED diet significantly modifies the gut microbiome, partially mediating cardiometabolic benefits. Supports dietary interventions targeting microbiome modulation.
Gao et al., 2024 [31]	Investigate how gut microbial metabolism of bile acids modifies effects of Med diet interventions on cardiometabolic risk.	18-month RCT (DIRECT-PLUS). 284 adults (88% male; mean age 51.2; BMI 31.2) with abdominal obesity/dyslipidemia.	All groups received physical activity guidance. Fecal bile acid profiling, gut microbiome sequencing, cardiometabolic biomarkers at baseline, 6, and 18 months. Diets: HDG, green-Med diet, Med diet.	BAs and risk: 14 fecal BAs associated with BMI and lipids. Med diet effects stronger in participants with lower 12-dehydrocholic acid or higher taurolithocholic acid sulfate. Gut microbes (e.g., Ruminococcus torques) modified bile acid-risk associations. Med diet reduced pro-inflammatory bile acids.	Fecal bile acids are biomarkers for predicting cardiometabolic risk and dietary responses. Med diet benefits are modulated by baseline bile acid profiles. Microbiome influences bile acid metabolism, offering probiotic/dietary intervention targets.
Ahrens et al., 2021 [32]	Evaluate effects of 6-day lifestyle-based immersion on cardiovascular risk factors and gut microbiota in adults at cardiometabolic risk.	Pilot study with pre-post analysis. 73 adults (mean age 46.9, 60.7% female) with moderate/high ASCVD risk.	Intervention: 6-day immersion program with 100% whole-food plant-based diet, daily exercise/yoga, stress management, and no caloric restriction.	Microbiota: ↑ butyrate producers (Lachnospiraceae +58.8%, Ruminococcaceae +82.1%, Faecalibacterium prausnitzii +54.5%). ↓ Bacteroides (-57.1%). Cardiometabolic: ↓ SBP (-4.46 mmHg), DBP (-3.38 mmHg), TC (-16.89 mg/dL), LDL (-9.82 mg/dL), TG (-24.81 mg/dL). Diversity: ↑ Shannon, Simpson indices.	Short-term lifestyle interventions may improve cardiovascular health by modulating gut microbiota. Supports plant-based diets and stress management for CVD prevention.
Ranaiyo et al., 2022 [33]	Investigate effects of chitin-glucan supplementation on postprandial metabolism and gut microbiota in subjects at cardiometabolic risk; assess exhaled H₂ as biomarker.	Double-blind, randomized, crossover study. 15 subjects (9 men, 6 women; mean age 44±10, BMI 28±2) with cardiometabolic risk.	CG arm: 4.5 g/day chitin-glucan (from Aspergillus niger). Control: 4.5 g/day maltodextrin. 3-day dietary records monitored; compliance 96%.	Postprandial metabolism: CG reduced iAUC for glycemia (-60 mM·min) and triglyceridemia (-35 mM·min) vs. control. Exhaled H₂ increased (+722 ppm·min). Microbiota: ↑ Erysipelotrichaceae UCG.003, Ruminococcaceae UCG.005, Eubacterium ventriosum; ↓ Actinobacteria family (p<0.05).	Supplementation improves postprandial glucose/lipid metabolism. Exhaled H₂ may predict responders to fiber interventions. Supports targeting gut microbiota for metabolic health.
Djekic et al., 2020 [34]	Investigate effects of vegetarian diet on cardiometabolic risk, gut microbiota, and plasma metabolome in IHD patients vs. isocaloric meat diet.	Randomized, open-label, crossover clinical trial. 31 subjects (94% men, median age 67) with IHD treated with PCI and optimal therapy.	Vegetarian diet: lacto-ovo-vegetarian diet with eggs/dairy, high plant foods. Meat diet: conventional diet with 145 g/day meat. Both are isocaloric, ready-made, and tailored.	Primary: Oxidized LDL-C lower with VD (-2.73 U/L, P=0.02). Secondary: lower TC (-5.03 mg/dL), LDL-C (-3.87 mg/dL), weight (-0.67 kg). No differences in HDL-C, TG, BP, and hs-CRP. Microbiota/metabolome: ↑ Akkermansia, ↓ Clostridium. ↓ L-carnitine (-14.77 µmol/L), TMAO trends.	VD may reduce cardiovascular risk beyond medical therapy. Ready-made VD meals improve adherence. Gut microbiota assessment may identify patients likely to benefit.

Glycemic control improvements: Numerous clinical trials have investigated the role of gut microbiota modulation, through probiotics, high-fiber diets, and plant-based nutritional strategies, in improving glycemic control among individuals with T2DM. Probiotic interventions, particularly those using strains like Lactobacillus acidophilus La-5 and Bifidobacterium animalis BB-12, have shown promising effects. Tonucci et al. (2016) reported a significant reduction in fructosamine levels (-9.91 mmol/L, p=0.04) and a borderline significant decrease in HbA1c (-0.67%, p=0.06), indicating better short-term and potential long-term glycemic control [20]. However, results across studies are not uniformly positive. For instance, Lestari et al. (2019) observed a significant reduction in fasting blood glucose (FBG) in the conventional yogurt group but not in the probiotic yogurt group, suggesting variability in effectiveness based on strain type, dosage, and patient characteristics. Diets that include a lot of fiber often demonstrate more regular improvements in blood sugar [22]. The study by Zhao et al. revealed that 89% of people who followed a high-fiber diet reached HbA1c levels lower than 7%, whereas only 50% in the control group did, and this was statistically significant (p<0.01) [23]. Chen et al. (2023) described enhanced FBG, HbA1c levels, and higher insulin and C-peptide, probably as a result of increased SCFAs in the gut, especially butyrate, which is linked to better insulin sensitivity and reduced inflammation [24]. In addition, following diets similar to the green-MED (Mediterranean) diet, as studied by Rinott et al. (2022), has been linked to better insulin resistance (homeostasis model assessment of insulin resistance (HOMA-IR)) and improved glucose regulation after meals, and some of these results are due to changes in microbes [30]. While FMT and probiotics were shown to benefit in several studies [1-7], Gómez-Pérez et al. (2024) found that FMT or probiotics did not significantly help control diabetes, indicating that microbiota management should be based on individual needs and is not simple [27].

Cardiometabolic risk reduction: Cardiometabolic risk includes several conditions, such as dyslipidemia, inflammation, obesity, and insulin resistance, which all raise the chance of having both cardiovascular disease and T2DM. Several recent clinical trials suggest that eating probiotics, increasing fiber in the diet, and adopting the MED way of eating may all benefit cardiometabolic markers through several linked benefits.

Lipid profile improvements: The study conducted by Ejtahed et al. in 2011 showed that daily eating of probiotic yogurt lowered low-density lipoprotein cholesterol (LDL-C) by 7.45% and total cholesterol (TC) by 4.54% in people with T2DM, proving the value of probiotics in controlling lipid metabolism [21]. Similar results were seen by Chen et al. in 2023, proving that increased fiber in the diet significantly decreased TC, LDL-C, and triglycerides and, at the same time, increased the protective high-density lipoprotein cholesterol (HDL-C) (p<0.05) [24]. This means that probiotics and dietary fibers can improve lipid levels, probably by helping bile acids to exit the body, making more SCFAs, and encouraging the activity of certain gut bacteria.

Anti-inflammatory effects: Cardiometabolic health is strongly affected by inflammation, and changing the microbiota seems to reduce inflammation. The study by Tonucci et al. (2016) revealed that probiotics reduced inflammation by decreasing TNF-α and resistin and helped keep the anti-inflammatory cytokine IL-10 continuous in the body, suggesting probiotics work on the immune system [20]. A similar study by Rinott et al. in 2022 found that sticking to a green-MED diet lowered levels of IL-1β, IL-6, and TNF-α (p<0.05), meaning that polyphenol-rich plant-based eating patterns help reduce inflammation through positive influence on gut bacteria [30].

Weight and metabolic syndrome markers: The green-MED diet yielded substantial weight loss (-6.5%) and significantly improved Framingham risk scores (q<0.001), indicating a comprehensive reduction in cardiometabolic risk. Moreover, Wan et al. (2019) found that low-fat diets enhanced populations of Blautia, a genus inversely correlated with LDL-C levels (r=-0.26, p<0.001), reinforcing the link between microbial diversity and metabolic health [25].

Novel Mechanisms

SCFAs and bile acids: Emerging evidence emphasizes the importance of microbiota-derived metabolites. Butyrate-producing bacteria such as Faecalibacterium have been associated with improved insulin sensitivity, providing mechanistic insights into microbial mediation of host metabolism. Gao et al. (2024) further showed that variations in bile acid metabolism, particularly levels of lithocholic acid and taurolithocholic acid sulfate, can predict individual responses to dietary interventions, paving the way for precision nutrition strategies based on gut microbial profiles [31].

Discussion

This systematic review shows that modifying gut microbiota with probiotics, high-fiber diets, and plant-based or MED eating plans can help improve glycemic control and reduce the risk of heart and blood sugar-related diseases in people with T2DM. Eating yoghurt, which has lots of probiotic bacteria, resulted in greater improvements than taking supplements, and having a high-fiber diet improved measures of HbA1c and lipids in Asian people. The study finds that SCFAs and bile acid signals in the gut help explain the differing effects of various diets. Still agreeing with earlier research, the fact that outcomes sometimes vary (e.g., the impact of FMT or changes in HDL-C) reveals how important it is to consider different microbial strains, different foods, and patients’ individual differences. These results suggest that T2DM should be managed by personalized ways of adjusting the gut microbiome and thorough studies into the mechanisms involved.

The findings of this systematic review agree with earlier studies about the role of gut microbiota in diabetes and heart disease (T2DM). As prior meta-analyses by Xu et al. in 2015 have shown, probiotics, especially those of Lactobacillus and Bifidobacterium, were able to reduce HbA1c and fasting glucose slightly [35]. Tonucci et al. (2016) noted that fructosamine, a time-averaged measure of blood glucose, had better results [20]. On the other hand, Lestari et al. (2019) did not find significant improvements in FBG levels, showing that the effects might vary depending on the strain or diet [22]. This review points out that choosing fermented dairy products (e.g., probiotic yoghurt) is better than settling for supplements in capsules.

People who followed high-fiber diets, especially prebiotic fiber diets, experienced better post-meal blood sugar. It was reported by Gurung et al. (2020) and Huda, Kim, and Bennett (2021) that diets like these help control HbA1c levels and increase Faecalibacterium and Roseburia groups of bacteria related to better insulin sensitivity. It is worth noting that by adding Asian groups to the study, there was a greater decrease in HbA1c with cultural diets like the WTP diet in China (89%) than without (50%) [36,37]. Chen et al. (2023) further found that SCFA production impacts the interactions between the gut and brain, which lowers diabetes-related depression [24].

According to Ejtahed et al. (2011), probiotic yoghurt was shown to lead to lower LDL cholesterol, and a healthy, high-fiber diet contributed to lower total and LDL cholesterol [21]. This study includes the fiber’s role in lowering bad cholesterol compared to good cholesterol, also called the TC: HDL-C ratio. It had been thought that probiotic strains alone influenced higher HDL-C through lipid benefits. Still, Lestari et al. (2019) proved that HDL-C increased with both probiotic and regular yoghurt, indicating that the fermentation of yoghurt may be beneficial [22]. Sharma et al. (2022) discovered that a low-fat diet encourages an increase in Blautia and may help reduce LDL-C, unlike earlier studies that tested high-fat diets [38].

Changing the microbiota had an impact on both inflammatory markers and oxidative stress. The study by Zhao et al. (2018) revealed that probiotics can lower the pro-inflammatory molecules TNF-α and resistin [23]. Rinott et al. (2022) found that following MED diets reduced IL-6 and CRP, and Wan et al. (2019) specifically associated this with a greater presence of Prevotella in the microbiome [25,30]. Some studies did not produce the same positive outcomes as expected. Even though Munoz et al. (2016) saw positive changes in metabolism after FMT, Gómez-Pérez et al. (2024) noted no differences in blood sugar after the same procedure, possibly because of variations in donor selection or the severity of T2D [27,39].

Weight management results and outcomes of metabolic syndrome were looked at as well. This study, Ramos-Romero et al. (2020), agreed with previous findings of Nie et al. (2019), noting that diets rich in polyphenols can help people lose weight (~6.5%) [28,40]. Amini-Salehi et al. (2024) noted the importance of eating high-quality fats, not only consuming less fat [41]. However, Djekic et al. (2020) discovered that vegetarian diets benefit LDL cholesterol regardless of changes in body mass index, but contrary to Ahrens et al. (2021), who found no change in cholesterol when people followed plant-based diets without weight loss [34].

Butyrate and SCFAs have been shown to help improve insulin sensitivity, as reported by Allin et al. (2015), while Chen et al. (2022) showed that these factors are also connected to better mood through the gut-brain axis [24,42]. On the other hand, Ramos-Romero et al. (2020) discovered that not all fibres are the same; grape pomace fibre did not influence gut bacteria, indicating that the source of fibre matters [28]. Studies by Gao et al. (2024) suggested that baseline bile acid patterns, including lithocholic acid, could better predict responses to specific diets, contrary to what Salgaço et al. (2019) previously suggested about TMAO [31,43].

All in all, probiotics are best consumed through fermented foods, with benefits depending on the specific organism; a high-fiber diet generally supports healthy blood sugar and lipids, with noticeable benefits observed in Asian studies; and eating mostly vegetables, cereals, fruits, and nuts from the MED diet or plant-based diets helps control weight and reduce inflammation. Recent findings on bile acids and phages may explain some differences in individual responses to treatment. The success of FMT trials appears to depend greatly on selecting the appropriate donor, the patient’s T2D status, and their baseline microbiota.

Clinical Implications

Gut microbiota-targeted interventions present promising adjunct strategies for managing T2DM. Personalized nutrition, tailored to individual microbiota profiles and dietary habits, may enhance glycemic control and metabolic health. Fermented probiotic-rich foods like yoghurt appear to be more clinically effective than capsule-based supplements due to better bioavailability and synergistic food matrix effects. High-fiber, prebiotic-rich diets consistently improve HbA1c and lipid parameters, offering a non-pharmacologic complement to standard T2D therapies. Furthermore, anti-inflammatory diets such as the MED and plant-based regimens contribute to reductions in systemic inflammation and cardiovascular risk. Emerging evidence also suggests gut-brain axis modulation through SCFAs could alleviate T2D-related depression and improve overall well-being.

This review has limitations of microbiota-targeted strategies for T2DM, including the heterogeneity of probiotic strains, fiber types, dietary protocols, sample sizes, and durations. Most trials were of short duration, limiting insights into long-term effectiveness and safety. Many studies also had relatively small sample sizes and high attrition rates, which may reduce statistical power and reliability. The findings were largely drawn from region-specific populations with limited ethnic diversity, restricting generalizability. Mechanistic exploration was limited in many studies, and conflicting results from FMT may be attributed to inconsistent protocols, donor variability, and differences in T2D progression among participants. Future research should prioritize long-term, large-scale RCTs to evaluate the sustainability and clinical impact of microbiota-targeted strategies on glycemic control, medication dependency, and complications. Personalized interventions guided by predictive biomarkers could revolutionize dietary and probiotic recommendations. Comparative effectiveness trials that directly evaluate probiotics, prebiotics, whole-diet interventions, and FMT will provide essential guidance for clinical decision-making.

## Conclusions

The study concluded that adherence to the green-Med diet led to significant improvements in body weight, insulin resistance, and Framingham risk scores. In addition, novel mechanisms were identified, particularly the role of SCFAs and bile acid metabolism as key modulators of host metabolic responses. These findings highlight the potential of microbiota-based interventions as promising strategies for improving glycemic control and reducing cardiometabolic risk in patients with T2DM.
